# On the Degrees of Freedom of Interference Alignment for Multicell MIMO Interfering Broadcast Channels

**DOI:** 10.1155/2014/827357

**Published:** 2014-02-10

**Authors:** Hyun-Ho Choi

**Affiliations:** Department of Electrical, Electronic, and Control Engineering and The Institute for Information Technology Convergence, Hankyong National University, Anseong 456-749, Republic of Korea

## Abstract

The interference alignment (IA) is a promising technique to efficiently mitigate interference and to enhance capacity of a wireless network. This paper proposes an interference alignment scheme for a cellular network with *L* cells and *K* users under a multiple-input multiple-output (MIMO) Gaussian interfering broadcast channel (IFBC) scenario. The proposed IA scheme aligns intercell interferences (ICI) into a small dimensional subspace through a cooperative receive beamforming and cancels both the ICI and interuser interferences (IUI) simultaneously through a transmit beamforming. We characterize the feasibility condition for the proposed IA to achieve a total number of degrees of freedom (DoF) of *LK* in terms of the numbers of transmit antennas and receive antennas. Then we derive the maximum number of DoF achieved by the proposed IA by finding an optimal dimension of ICI alignment subspace for a given antenna configuration. The numerical results show that the proposed IA scheme has a better DoF performance than the conventional schemes.

## 1. Introduction

Interference management is a key challenge in the design of the current and future cellular networks in order to satisfy the demand for higher data rates. To increase the system capacity in multicell and multiuser wireless network environments, various interference management techniques based on coordinated multipoint transmission and reception have been presented [1–3]. Among them, an interference alignment (IA) technique has been recently highlighted as an efficient capacity-achieving interference management scheme [4]. The basic idea of interference alignment is to align the interference signals in a reduced dimensional subspace at each receiver so that an interference-free orthogonal subspace can be solely allocated for data transmission. To date, various interference alignment algorithms have been proposed and analyzed in X network [5–7], multiple-input multiple-output (MIMO) interference network [8–10], and cellular network [11–16].

In the multi-cell MIMO Gaussian interfering broadcast channels (MIMO-IFBC), each base station (BS) supports multiple users within its cell and so there exist two kinds of interference, namely, interuser interference (IUI) and intercell interference (ICI). To mitigate both IUI and ICI simultaneously, a simple zero-forcing (ZF) scheme has been proposed for two-cell MIMO-IFBC [11]. This ZF scheme achieves a number of degrees of freedom (DoF) of min⁡{2*M*, 2*KN*, max⁡(*M*, *N*)} when there are two BSs with *M* transmit antennas and *K* users with *N* receive antennas in each cell. Moreover, a precoding scheme called subspace interference alignment for two-cell MIMO-IFBC has been proposed [13]. This subspace IA consists of two cascaded precoders. The first precoder puts the ICI vectors into a finite multidimensional subspace and the second precoder makes the IUI vectors lie on the subspace spanned by the ICI vectors. This increases the dimension of subspace for the desired signal vectors, especially in the case of a symmetric antenna configuration. There are further results on the achievable DoF obtained by IA in the two-cell case [14–16]. Authors in [14] showed that the DoF per cell is achieved as *KM*/(*K* + min⁡(*K*, *M*)) by assuming time or frequency extension in two-cell multiple-input single-output (MISO) IFBC. Authors in [16] proved that *K* DoF per cell can be achieved when *M* = *K* + 1 and *N* = *K* in two-cell *K*-user MIMO-IFBC. Authors in [15] presented the optimality of IA by providing a tight DoF outer bound in the two-cell MIMO network. Furthermore, an IA solution in the three-cell case has been proposed [12]. This IA algorithm jointly designs transmit and receive beamforming matrices based on user cooperation. Recently, the interference channel alignment scheme has been presented [17] and becomes the basis for this study.

The previous studies have mainly considered two- or three-cell case and there is still no closed-form solution for a general multi-cell case. In this paper, we propose a new IA scheme for a general *L*-cell and *K*-user MIMO-IFBC scenario. The proposed IA scheme aligns multiple ICIs into a small dimensional subspace through a cooperative receive beamforming and cancels both the ICI and IUI simultaneously through a transmit beamforming. We characterize the feasibility condition for the proposed IA to achieve a total DoF of *LK* in terms of the numbers of transmit antennas and receive antennas. Then, we derive the maximum number of DoF achieved by the proposed IA by finding an optimal dimension of ICI alignment subspace for a given antenna configuration.

The rest of this paper is organized as follows. In Section 2, we describe a system model for the considered multi-cell MIMO-IFBC. In Section 3, we explain the operation of the proposed IA in detail. In Sections 4 and 5, we investigate the feasibility and the achievability of the proposed IA, respectively. In Section 6, we compare the proposed IA with the conventional schemes in terms of the achievable DoF. Finally, we conclude this paper in Section 7.

## 2. System Model

**Figure 1:**
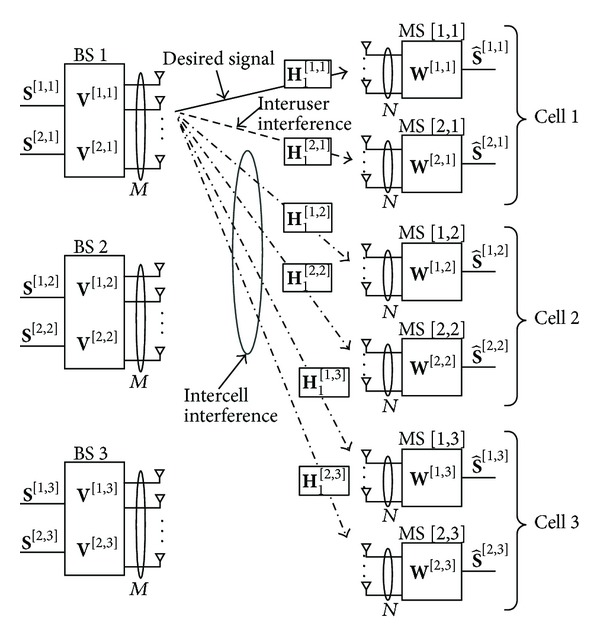
MIMO-IFBC for the case of three cells and two users per cell (*L* = 3 and *K* = 2).

We describe a system model for the considered multi-cell MIMO-IFBC scenario. The system contains *L* cells. Each cell has one BS and *K* users, that is, mobile stations (MS). We denote the *k*th MS in the *i*th cell as [*k*, *i*] for *k* ∈ {1,2,…, *K*} and *i* ∈ {1,2,…, *L*}. We assume that each BS is equipped with *M* antennas and each MS is equipped with *N* antennas. An example for the case of *L* = 3 and *K* = 2 is illustrated in Figure 1. We define some parameters and notations as follows:

- **H**_*j*_^[*k*,*i*]^: *N* × *M* channel matrix from the BS *j* to the MS [*k*, *i*], of which entry is independent and identically distributed (i.i.d.) with *𝒞𝒩*(0,1);
- *s* ^[*k*,*i*]^: symbol transmitted to the *k*th MS in the *i*th cell with an average power constraint *P*;
- **v** ^[*k*,*i*]^: *M* × 1 transmit beamforming vector for carrying the symbol *s* ^[*k*,*i*]^ with the unit norm ||**v** ^[*k*,*i*]^|| = 1;
- **w** ^[*k*,*i*]^: *N* × 1 receive beamforming vector at the MS [*k*, *i*] with the unit norm ||**w** ^[*k*,*i*]^|| = 1;
- **n** ^[*k*,*i*]^: *N* × 1 additive white Gaussian noise (AWGN) vector at the MS [*k*, *i*] with variance *σ* ^2^ per entry;
- (·)^†^: conjugate transpose operator.

We suppose that each BS tries to convey one data stream per MS. Therefore, the received signal **y** ^[*k*,*i*]^ at the MS [*k*, *i*] is represented as

(1) y[k,i]=Hi[k,i]v[k,i]s[k,i]︸desired  signal+∑k′=1,k′≠kKHi[k,i]v[k′,i]s[k′,i]︸interuser  interference +∑i′=1,i′≠iL ∑k′=1KHi′[k,i]v[k′,i′]s[k′,i′]︸intercell  interference+n[k,i].

After each MS decodes its desired signal by applying receive beamforming, the signal at the MS [*k*, *i*] is expressed as

(2) y~[k,i]=w[k,i]†Hi[k,i]v[k,i]s[k,i] +w[k,i]†∑k′=1,k′≠kKHi[k,i]v[k′,i]s[k′,i] +w[k,i]†∑i′=1,i′≠iL ∑k′=1KHi′[k,i]v[k′,i′]s[k′,i′]+n~[k,i],

where ${\tilde{n}}^{\left[k,i\right]}=w^{[k,i]†}n^{\left[k,i\right]}$ is the effective noise. Thus, the achievable rate at the MS [*k*, *i*] is expressed as

(3) R[k,i] =log⁡2⁡(1+(|w[k,i]†Hi[k,i]v[k,i]|2P)×(∑k′=1,k′≠kK|w[k,i]†Hi[k,i]v[k′,i]|2P+∑i′=1,i′≠iL ∑k′=1K|w[k,i]†Hi′[k,i]v[k′,i′]|2P +σ2)−1).

We define the DoF (a.k.a. multiplexing gain) for our multi-cell network as the prelog factor of the sum rate [8]. This is one of the key metrics used for assessing the performance of a multiple antenna-based system at high SNR regime. Therefore, the individual DoF achieved by MS [*k*, *i*] and the total DoF are, respectively, expressed as

(4) $$\begin{array}{c} d^{\left[k,i\right]}≜\underset{\text{SNR}\to \infty }{\lim}\frac{R^{\left[k,i\right]}\left(\text{SNR}\right)}{\log\left(\text{SNR}\right)},\;\;d_{\Sigma }=\sum_{k=1}^{K}\;\sum_{i=1}^{L}d^{\left[k,i\right]}, \end{array}$$

where the SNR is given by *P*/*σ* ^2^.

## 3. Proposed Interference Alignment Scheme

**Figure 2:**
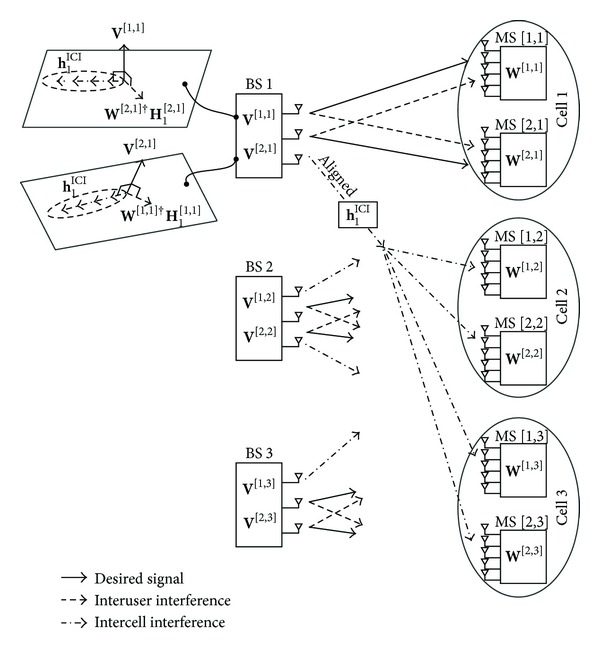
Proposed IA scheme for (*L*, *K*, *M*, *N*) = (3,2, 3,5).

The proposed IA scheme operates with two steps. First, each MS cooperatively constructs the receive beamforming vectors to align the effective ICI channel within a small dimensional subspace. Through this ICI channel alignment, each BS can regard *K*(*L* − 1) different ICI channels as Δ-dimensional ICI channel vectors with Δ < *K*(*L* − 1). Then, each BS removes both ICI and IUI simultaneously by making the transmit beamforming vector orthogonal to the subspace spanned by the effective ICI channel and IUI channel vectors. For this operation, we assume that the perfect knowledge of channel state information (CSI) is available at both the BS and the MS. To facilitate understanding of the proposed IA scheme, we first explain it with a motivating example for (*L*, *K*, *M*, *N*) = (3,2, 3,5), as illustrated in Figure 2. Thereafter, we extend it to the generalized case and discuss its feasibility.

**Step 1 (design of receive beamforming vectors through ICI channel alignment)**

The receive beamforming vectors of MS [1, *i*] and MS [2, *i*], **w** ^[1,*i*]^ and **w** ^[2,*i*]^, are determined in order that the effective ICI channels from the BS *i* to all nonintended MSs in the other cell are aligned, where *i* ∈ {1,2, 3}. The ICI channel alignment conditions in each BS are given by

(5) span⁡(h1ICI)=span⁡(H1[1,2]†w[1,2])=span⁡(H1[2,2]†w[2,2])=span⁡(H1[1,3]†w[1,3])=span⁡(H1[2,3]†w[2,3]),span⁡(h2ICI)=span⁡(H2[1,1]†w[1,1])=span⁡(H2[2,1]†w[2,1])=span⁡(H2[1,3]†w[1,3])=span⁡(H2[2,3]†w[2,3]),span⁡(h3ICI)=span⁡(H3[1,1]†w[1,1])=span⁡(H3[2,1]†w[2,1])=span⁡(H3[1,2]†w[1,2])=span⁡(H3[2,2]†w[2,2]),

where span⁡(·) means the space spanned by the column vectors of a matrix and **h**_*i*_^ICI^ means the basis vector of aligned effective interference channels after applying the receive beamforming vectors to all the interfered MSs that suffer from ICI by the interfering BS *i*. With more restrictive conditions, (5) can be rewritten as

(6) h1ICI=H1[1,2]†w[1,2]=H1[2,2]†w[2,2]=H1[1,3]†w[1,3]=H1[2,3]†w[2,3],h2ICI=H2[1,1]†w[1,1]=H2[2,1]†w[2,1]=H2[1,3]†w[1,3]=H2[2,3]†w[2,3],h3ICI=H3[1,1]†w[1,1]=H3[2,1]†w[2,1]=H3[1,2]†w[1,2]=H3[2,2]†w[2,2].

To jointly construct the received beamforming vectors, we aggregate the ICI channel alignment conditions of all BSs given by (6) into a unified system equation, which is expressed as

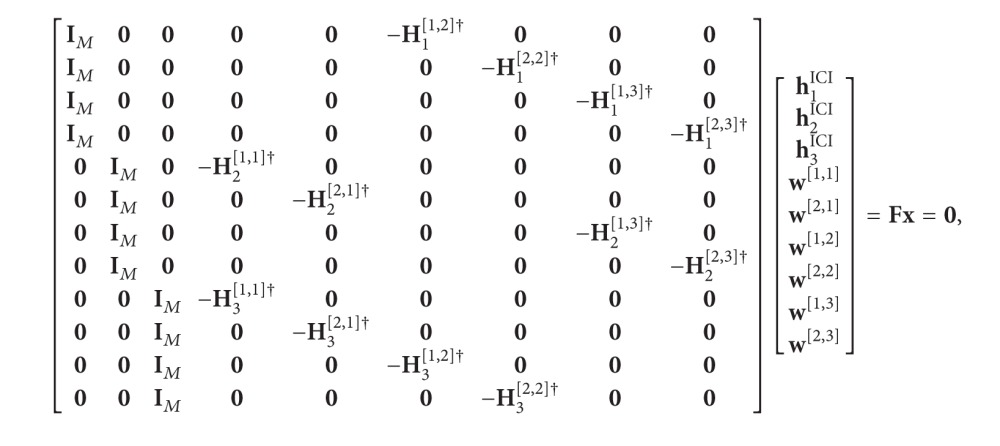
(7)
where **F** is a 12*M* × (3*M* + 6*N*) matrix with orthogonal columns. By finding the null space of **F**, we can obtain the receive beamforming vectors that satisfy (6). There exists a vector **x** in the null space of **F** only if the column size of **F** is greater than the row size of **F** when the DoF of each MS is one. Therefore, the condition 3*M* + 6*N* > 12*M* should be satisfied. In this case for *M* = 3 and *N* = 5, the size of **F** is 36 × 39. Thus, it has a three-dimensional null space and so the receive beamforming vectors for ICI channel alignment are obtained explicitly with probability one. For any given *L*, *K*, *M*, *N*, and Δ, the existence condition of the null space of **F** will be discussed in Section 4.

**Step 2 (design of transmit beamforming vectors through IUI and ICI cancellations)**

Since the effective ICI channels are aligned with each other, the BS *i* can regard four different ICI channel vectors as a single ICI channel vector that spans one-dimensional subspace, as shown in Figure 2. Therefore, the BS *i* can send the symbols *s* ^[1,*i*]^ and *s* ^[2,*i*]^ to the MS [1, *i*] and MS [2, *i*], respectively, by using only three transmit antennas without any interference. To completely eliminate interferences, the transmit beamforming vectors for two MSs in the BS *i*, **v** ^[1,*i*]^ and **v** ^[2,*i*]^, are, respectively, determined as

(8) $$\begin{array}{c} v^{\left[1,i\right]}\subset \text{null}\left({\left[{\left(w^{[2,i]†}H_{i}^{\left[2,i\right]}\right)}^{†},h_{i}^{\text{ICI}}\right]}^{†}\right), \end{array}$$

(9) $$\begin{array}{c} v^{\left[2,i\right]}\subset \text{null}\left({\left[{\left(w^{[1,i]†}H_{i}^{\left[1,i\right]}\right)}^{†},h_{i}^{\text{ICI}}\right]}^{†}\right), \end{array}$$

where null(·) means an orthonormal basis for the null space of a matrix.

The received beamforming vector **w** derived from (7) and the transmit beamforming vector **v** obtained by (8) and (9) can remove both ICI and IUI simultaneously. Thus, if these **w** and **v** are applied to (3), both the ICI term ∑_*i*′=1,*i*′≠*i*_^*L*^∑_*k*′=1_^*K*^|**w** ^[*k*,*i*]†^ **H**_*i*′_^[*k*,*i*]^ **v** ^[*k*′,*i*′]^|^2^ and the IUI term ∑_*k*′=1,*k*′≠*k*_^*K*^|**w** ^[*k*,*i*]†^ **H**_*i*_^[*k*,*i*]^ **v** ^[*k*′,*i*]^|^2^ approach zero. Therefore, the achievable rate at the MS [*k*, *i*] in the proposed IA scheme can be reexpressed as

(10) $$\begin{array}{c} R_{\text{proposed}}^{\left[k,i\right]}\approx \log_{2}\left(1+\frac{{\left|w^{[k,i]†}H_{i}^{\left[k,i\right]}v^{\left[k,i\right]}\right|}^{2}P}{\sigma^{2}}\right). \end{array}$$

That is, the proposed IA improves the achievable rate and this improved rate accordingly increases the achieved DoF performance given by (4).

## 4. Feasibility of Proposed IA

In this section, we extend the proposed IA scheme to the general case for *L* cells and *K* users and derive its feasibility condition, based on the result of previous exemplary case. The feasibility condition of the proposed IA is summarized as follows.

**Theorem 1**

For the *L*-cell and *K*-user MIMO-IFBC with *M* transmit antennas and *N* receive antennas, one DoF per user (i.e., total *LK* DoFs) is achieved if

(11) $$\begin{array}{c} M\geq K+\Delta ,\;\;N>\frac{\left\{\left(L-1\right)K-\Delta \right\}M}{K}, \end{array}$$

where Δ ∈ {0,1,…, min⁡[(*L* − 1)*K*, *M*] − 1} denotes the dimension of the aligned effective ICI channels.

**Proof**

To align *K*(*L* − 1) effective ICIs within Δ-dimensional subspace, the receive beamforming vectors should be constructed as

(12) span⁡([hj,1ICI,hj,2ICI,…,hj,ΔICI]) =span⁡([HjICI,1,…,HjICI,j−1,HjICI,j+1,…,HjICI,L]),

where **H**_*j*_^ICI,*i*^ = [**H**_*j*_^[1,*i*]†^ **w** ^[1,*i*]^, **H**_*j*_^[2,*i*]†^ **w** ^[2,*i*]^,…, **H**_*j*_^[*K*,*i*]†^ **w** ^[*K*,*i*]^] is a channel matrix composed of *K* ICI vectors from the *j*th BS to all *K* MSs in the *i*th cell after applying the receive beamforming vectors for *i* ≠ *j*. To minimize the required number of transmit and receive antennas, we need to restrict condition (12) and can represent it as a matrix form in the same way as (7). Thereafter, we can generate a new unified system matrix **F** with a size of *L*(*L* − 1)*MK* × (*LM*Δ + *L* *KN*) and obtain the receive beamforming vectors by finding the null space of **F**.

The existence condition of the null space of **F** is given by

(13) (LMΔ+LKN)>L(L−1)MK

(14) ⟹N>{(L−1)K−Δ}MK.

Because *N* > 0, the dimension Δ should satisfy Δ < (*L* − 1)*K*. In addition, since all the effective ICI channels after applying receive beamforming vectors are aligned within a Δ-dimensional subspace, the number of transmit antennas required to eliminate both ICI and IUI simultaneously is given by

(15) $$\begin{array}{c} M\geq K+\Delta . \end{array}$$

Because *K* > 0, the dimension Δ should also satisfy Δ < *M*. Therefore, two conditions, Δ < (*L* − 1)*K* and Δ < *M*, and (14) and (15) prove Theorem 1.

**Remark 2**

When the number of cells is two (i.e., *L* = 2) and the dimension for ICI alignment is one (i.e., Δ = 1), the derived feasibility condition (11) is reduced as

(16) M≥K+1,  N>(K−1)MK=(K−1)(K+1)K⟹M≥K+1,  N≥K.

This required antenna configuration is equivalent to the result in [16]. Moreover, the result in [15] indicates that, in this two-cell case, the proposed IA scheme achieves the optimal DoF.

## 5. Achievability of Proposed IA

From a practical point of view, the cellular system has a limited number of antennas due to the lack of physical space in equipments, but the number of cooperative cells or users is variable according to the geographical coverage and the scheduling method. In this context, we can verify the achievability of the proposed IA by maximizing the number of users per cell while each user has one DoF. Therefore, in this section, we aim to maximize the value of *K* by choosing an optimal dimension Δ for the ICI alignment at a given (*L*, *M*, *N*) condition.

Two conditions in (11) are combined as

(17) $$\begin{array}{c} \frac{KN}{\left(L-1\right)K-\Delta }>M\geq K+\Delta . \end{array}$$

From *M* ≥ *K* + Δ and *KN*/((*L* − 1)*K* − Δ) > *K* + Δ, we can obtain two conditions for *K*, respectively, as follows:

(18) $$\begin{array}{c} K\leq M-\Delta ,\\ K<\frac{N-\left(L-2\right)\Delta +\sqrt{{\left\{N-\left(L-2\right)\Delta \right\}}^{2}+4\left(L-1\right)\Delta^{2}}}{2\left(L-1\right)}. \end{array}$$

Accordingly, *K* is bounded as

(19) K≤min⁡{M−Δ,⌊N−(L−2)Δ2(L−1)+{N−(L−2)Δ}2+4(L−1)Δ22(L−1)⌋}

(20)  ⟹K≤min⁡{f1(Δ),⌊f2(Δ)⌋},

where we define two functions as *f*_1_(Δ) = *M* − Δ and $f_{2}(\Delta )=\left(N-\left(L-2\right)\Delta +\sqrt{{\left\{N-\left(L-2\right)\Delta \right\}}^{2}+4\left(L-1\right)\Delta^{2}}\right)/2\left(L-1\right)$ according to Δ ∈ {0,1,…, min⁡[(*L* − 1)*K*, *M*] − 1}.

Our objective is to maximize *K* according to Δ at a given (*L*, *M*, *N*) condition. Therefore, the optimization problem is formulated as

(21) max⁡Δ K=max⁡Δmin⁡{f1(Δ),⌊f2(Δ)⌋}s.t. L,M,N∈{2,3,4,…},  Δ∈{0,1,…,M−1}.

Notice that *f*_1_ is a decreasing function for Δ and *f*_2_ is a convex function for Δ because *d* ^2^ *f*_2_/*d*Δ^2^ = 2*N* ^2^/{4*N*Δ+(*N*−*L*Δ)^2^}^3/2^ > 0. In addition, by calculating *f*_1_(Δ_*c*_) = *f*_2_(Δ_*c*_), we know that *f*_1_ and *f*_2_ are crossed at Δ_*c*_ = ((*L* − 1)*M* ^2^ − *MN*)/(*LM* − *N*) < *M* within the available range of Δ. Thus, the maximum value of *K* (i.e., max⁡ min⁡{*f*_1_(Δ), ⌊*f*_2_(Δ)⌋}) can be found at either Δ = 0 or Δ = Δ_*c*_. From (19), a local maximum *K*_1_ at Δ = 0 is given by

(22) $$\begin{array}{c} K_{1}=\min\left\{M,\left\lfloor \frac{N}{L-1}\right\rfloor \right\}. \end{array}$$

Also, the other local maximum *K*_2_ at Δ = Δ_*c*_ is given by

(23) K2=⌊f2(Δc)⌋=⌊f1(Δc)⌋=⌊M−(L−1)M2−MNLM−N⌋=⌊M2LM−N⌋.

Consequently, the maximum *K* value, K*, is obtained as

(24) K∗=max⁡{K1,K2}=max⁡{min⁡{M,⌊NL−1⌋},⌊M2LM−N⌋}.

Notice that *K*_1_ = *K*_2_ if *M* = *N*.

Therefore, the achievability of the proposed IA scheme is summarized as follows.

**Theorem 3**

For the *L*-cell and *K*-user MIMO-IFBC with *M* transmit antennas and *N* receive antennas, if *M* ≥ *N*, the maximum DoF per cell (i.e., maximum *K*) is achieved as

(25) $$\begin{array}{c} K^{*}=\left\lfloor \frac{M^{2}}{LM-N}\right\rfloor \;at\;\Delta^{*}=\left\lfloor \frac{\left(L-1\right)M^{2}-MN}{LM-N}\right\rfloor , \end{array}$$

where Δ* is the optimal dimension parameter for the ICI alignment. On the other hand, if *M* < *N*, the maximum DoF per cell is achieved as

(26) $$\begin{array}{c} K^{*}=\min\left\{M,\left\lfloor \frac{N}{L-1}\right\rfloor \right\}\;at\;\Delta^{*}=0. \end{array}$$

**Remark 4**

When the number of transmit antennas is less than the number of receive antennas (i.e., *M* < *N*), the optimal dimension for ICI alignment is zero (i.e., Δ* = 0). In this case, the proposed IA scheme is equivalent to a multi-user MIMO scheme [18]. Namely, each BS deals with only the IUI, and each user eliminates the remaining ICIs as it decodes all received signals and discards out-of-cell users' signals.

## 6. Result and Discussion

We compare the proposed IA scheme with the orthogonalization (i.e., resource partitioning among BSs), subspace IA [13], three-cell IA [12], and outer bound [19]. For the proposed IA, we consider the number of cooperating cells, *L* ∈ {2,3, 6}. Note that the subspace IA can achieve interference-free DoF only for the two-cell case (i.e., *L* = 2) and its total DoF is given by 2{min⁡(*M*, *N*) − 1}. On the other hand, the three-cell IA is available only for the three-cell case (i.e., *L* = 3) and its total DoF is given by 3*Kd* where *d* ≤ ⌊*M*/(3*K* − 1)⌋ if *M* = *KN* and *d* ≤ min⁡{⌊*M*/(3*K* − 1)⌋, 3(*KN* − *M*)} if *M* < *KN*.

**Figure 3:**
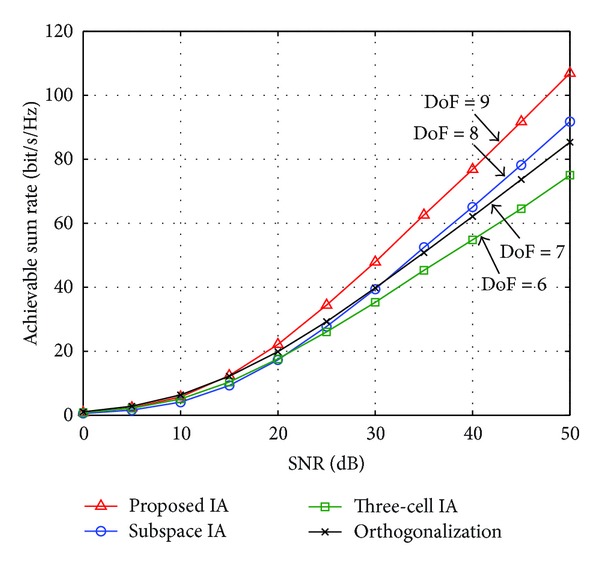
Ergodic sum rate versus SNR for (*L*, *K*, *M*, *N*) = (3,3, 7,5).

Figure 3 shows the ergodic sum rate as a function of SNR in the case of (*L*, *K*, *M*, *N*) = (3,3, 7,5). As shown, the proposed IA scheme outperforms the other schemes. The increasing rate of the proposed IA scheme according to the SNR (i.e., DoF) is 9 while that of the other schemes is less than 9. That is, the proposed IA scheme improves the sum rate performance by increasing the DoF.

**Figure 4:**
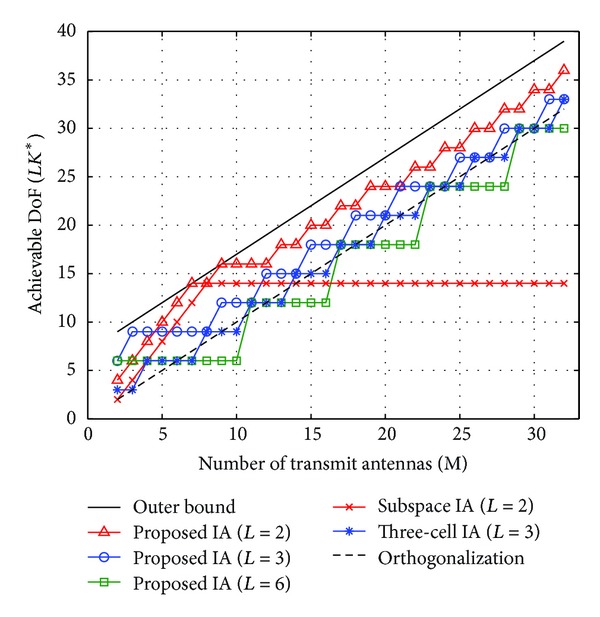
Achievable DoF versus number of transmit antennas when *N* = 8.

Figure 4 shows the achievable DoF versus the number of transmit antennas (*M*) when *N* is fixed to 8. As *M* increases, the achievable DoF of both the proposed IA and three-cell IA schemes increases, but that of the subspace IA is saturated soon because its maximum DoF is limited by the minimum value of *M* and *N*. As *L* increases, the performance of the proposed IA decreases because the number of ICIs increases. However, the proposed IA scheme shows better DoF performances than the conventional schemes when *L* ≤ 3. This is attributed to the fact that the proposed IA searches for the optimal dimension of ICI alignment subspace and exploits the signal space more efficiently.

**Figure 5:**
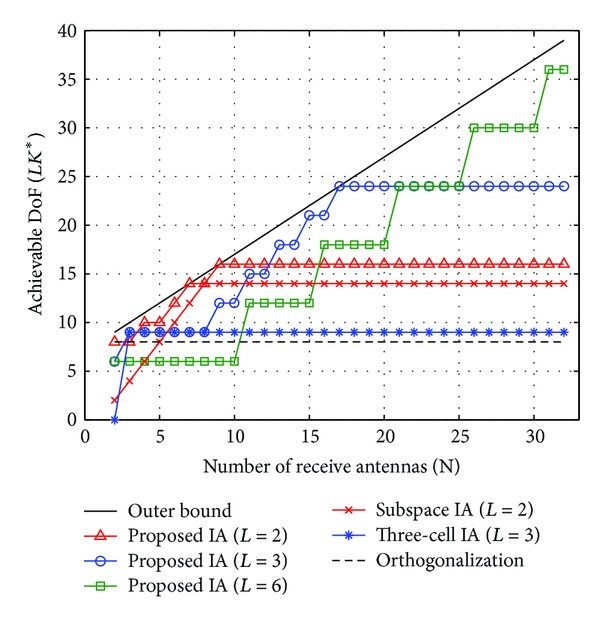
Achievable DoF versus number of receive antennas when *M* = 8.

Figure 5 shows the achievable DoF versus the number of receive antennas (*N*) when *M* is fixed to 8. Similarly, the proposed IA scheme outperforms the corresponding conventional scheme in each *L* value. Compared to the result of Figure 4, when the number of receive antennas is increased, the achievable DoF is increased but eventually saturated. This is because the achievable DoF of the proposed IA is limited to *LM* when *M* < *N* according to the result of (26). Similar to the result in Figure 4, the DoF of the subspace IA is limited by min⁡(*M*, *N*). Moreover, the DoF of the three-cell IA is limited by ⌊*M*/(3*K* − 1)⌋ when *M* is fixed, and the DoF of the orthogonal scheme is limited by the number of transmit antennas *M*. From two results of Figures 4 and 5, we can choose appropriate parameters, *L*, *M*, and *N*, to achieve maximum DoF.

## 7. Conclusion

We proposed an IA scheme for the multi-cell MIMO-IFBC by jointly designing the transmit and receive beamforming vectors using a closed-form expression. The proposed IA scheme aligns multiple ICIs into a small dimensional subspace through the receive beamforming and removes both the ICI and IUI simultaneously through the transmit beamforming. As the feasibility condition of the proposed IA, we derived the minimum number of antennas required to achieve one DoF per user. For the achievability of the proposed IA, we derived the maximum number of supportable users for a given antenna configuration. The numerical results showed that the proposed IA scheme has a better DoF performance than the conventional schemes and informed how to determine the related parameters to achieve maximum DoF.
